# Supplementary material for: Antioxidant Potential and Cytotoxic Effect of Isoflavones Extract from Thai Fermented Soybean (Thua-Nao)
Source: Molecules. 2021 Dec 8;26(24):7432. doi: 10.3390/molecules26247432 (PMC8705088; doi:10.3390/molecules26247432)
Supplement: Supplementary file 1 [file molecules-26-07432-s001.zip › molecules-1492563-supplementary.pdf]

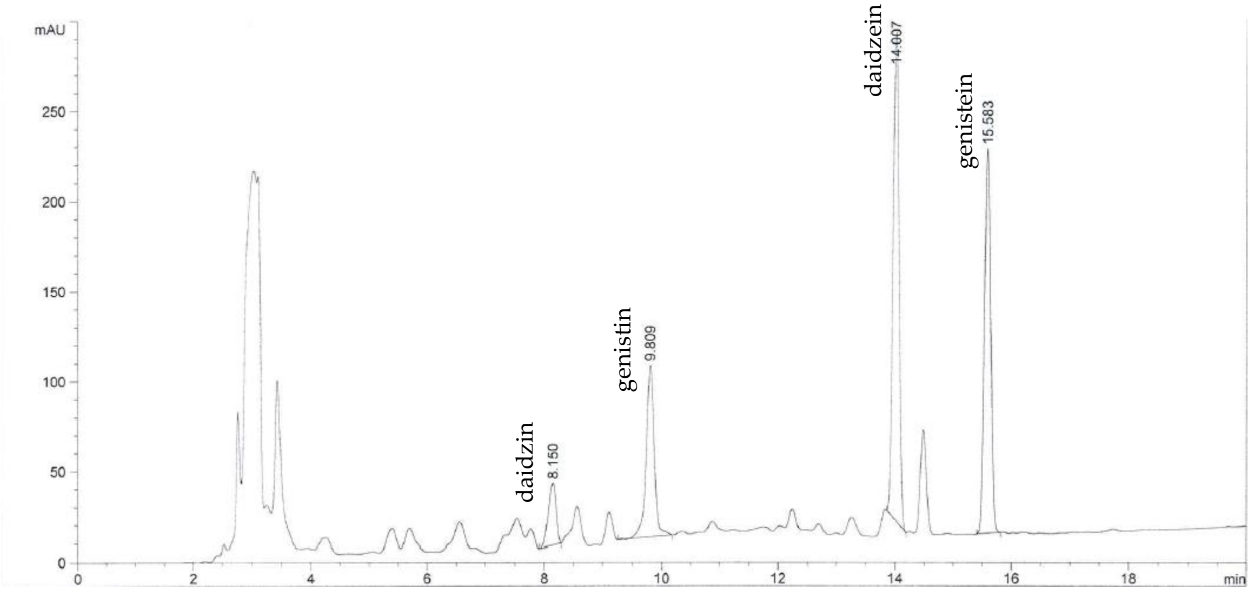

Figure S1: HPLC chromatograms of the ethanolic isoflavones extract from Thai fermented soybean (Thua-nao) incubated with Bacillus species during fermentation.

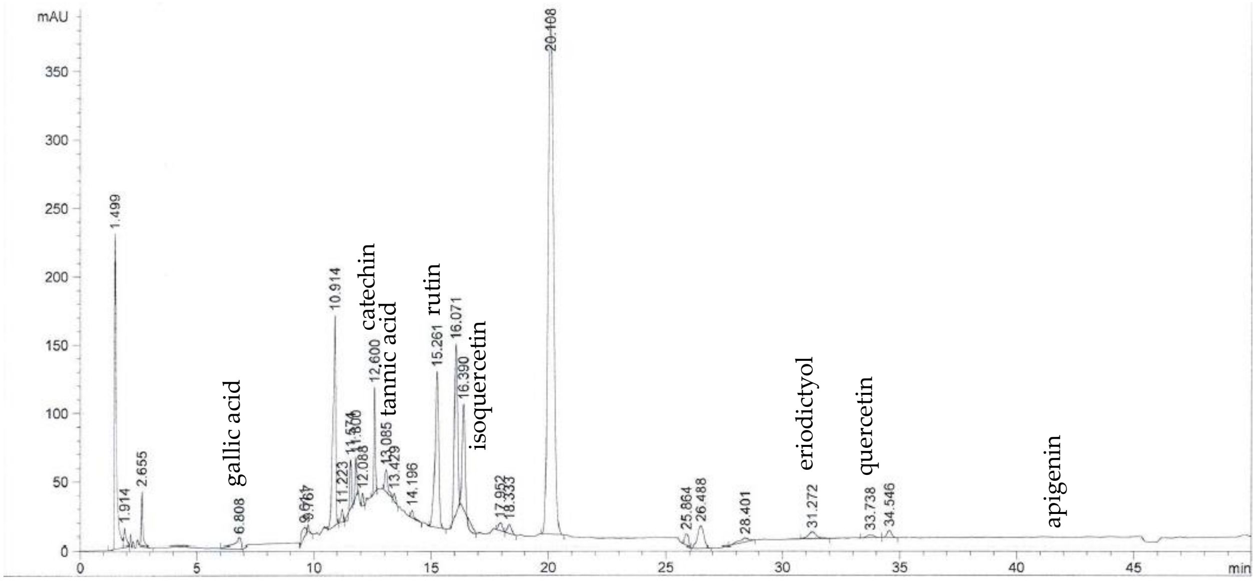

Figure S2: Chromatograms of different of phenolic compounds from the ethanolic isoflavones extract from Thai fermented soybean (Thua-nao) obtained by the HPLC method.
